# Design and simulation of 4 kW solar power-based hybrid EV charging station

**DOI:** 10.1038/s41598-024-56833-5

**Published:** 2024-03-27

**Authors:** Priyanshu Singla, Shakuntla Boora, Poonam Singhal, Nitin Mittal, Vikas Mittal, Fikreselam Gared

**Affiliations:** 1 Department of Electrical Engineering, J. C. Bose University of Science and Technology YMCA, Faridabad, India; 2 Skill Faculty of Engineering and Technology, Shri Vishwakarma Skill University, Palwal, Haryana 121102 India; 3 Department of Electronics and Communication Engineering, Chandigarh University, Mohali, Punjab 140413 India; 4 Faculty of Electrical and Computer Engineering, Bahir Dar University, Bahir Dar, Ethiopia

**Keywords:** Hybrid electric vehicles, Solar power, P&O algorithm, PVsyst, Electric vehicles battery charging station, Energy science and technology, Engineering

## Abstract

Electric vehicles (EVs) have become an attractive alternative to IC engine cars due to the increased interest in lowering the consumption of fossil fuels and pollution. This paper presents the design and simulation of a 4 kW solar power-based hybrid EV charging station. With the increasing demand for electric vehicles and the strain they pose on the electrical grid, particularly at fast and superfast charging stations, the development of sustainable and efficient charging infrastructure is crucial. The proposed hybrid charging station integrates solar power and battery energy storage to provide uninterrupted power for EVs, reducing reliance on fossil fuels and minimizing grid overload. The system operates using a three-stage charging strategy, with the PV array, battery bank, and grid electricity ensuring continuous power supply for EVs. Additionally, the system can export surplus solar energy to the grid, reducing the load demand. The paper also discusses the use of MPPT techniques, PV cell modeling, and charge controller algorithms to optimize the performance of the hybrid charging station.

## Introduction

The need for fuels is great in the current situation, and their consumption rises. These fuels’ usage in automobiles caused a significant quantity of CO_2_ petrol to evaporate. The environment’s response to carbon dioxide gas varies greatly. The biggest difficulty is reducing CO_2_, and an eco-friendly car, sometimes known as an electric vehicle (EV), can help. In the age of e-mobility, consumers are being encouraged to switch quickly to EVs, but widespread adoption of EVs into the electrical grid, particularly at fast and superfast charging stations, could put a significant strain on the stability and dependability of the grid, with peak demand overload, voltage sag, and power gaps being the main problems^1^. For overcoming these problems Renewable energy and battery energy storage (BESS) are good options to replace traditional charging stations with hybrid charging stations which provide uninterrupted power for electric vehicles. Solar photovoltaic systems involve the direct conversion of sunlight into electricity without affecting the environment. In recent years, it has been observed that the use of electric vehicles in the market has increased and charging these vehicles has become a difficult task for passengers. Photovoltaic plants have also become cheaper in recent years and have proven to be an effective way of generating electricity^2^.

The power converter must be between source and load. Therefore, the use of renewable energy and battery bank power has increased^3^. The main purpose of this project is to charge electric vehicles using BES and solar power. Solar PV panels and battery energy storage systems (BES) create charging stations that power EVs. AC grids are used when the battery of the solar power plant runs out or when weather conditions are not appropriate.

In addition, charging stations can facilitate active/reactive power transfer between battery and grid, as well as vehicle. During the day, the photovoltaic array produces enough electricity to charge the battery of an electric car. When the sun is at its peak, the PV array not only charges the EV battery but also feeds back additional energy into the single-phase grid system. When the sun is not shining or the sky is dark, the EV battery will be charged by the battery bank and grid also. The Perturb & Observe (P&O) algorithm based MPPT controller used in the closed loop of this project to provide peak power at constant voltage and a bidirectional buck/boost converter with inverter connected to single phase AC grid was designed using MATLAB simulation.

### Motivation

The combination of solar power and EV charging is crucial to reducing our reliance on fossil fuels. Electricity comes from many sources and it is important that the electric car be powered by renewable energy. Electric cars are becoming very popular, and we expect almost everyone who owns a solar panel to have a solar charging station in their home in the next few years. Grid-connected PV arrays offer optimal EV charging by synchronizing with daily energy demand profiles. Surplus photovoltaic generation during peak solar hours seamlessly integrates into the utility grid, enabling net metering benefits even during car usage. Upon returning home, the accumulated credit offsets electric vehicle charging through bidirectional power flow, effectively leveraging home-generated solar for EV transportation.

## Literature review

Patel^4^ has stated that the intermittent nature of the PV output power makes it weather-dependent. In a fast-charging station powered by renewable energy, the battery storage is therefore paired with a grid-tied PV system to offer an ongoing supply for on-site charging of electric vehicles. In order to support the high charging rates needed for connecting a significant number of EVs to the grid, fast charging stations based on renewable energy should be affordable, effective, and dependable.

Tan^5^ has suggested a better design in which the charge controller is implemented using a buck converter acting as a DC-DC converter. The Perturb and Observe (P&O) MPPT algorithm keeps track of the photovoltaic panel’s maximum output. The lithium-ion battery is charged by the battery charge controller in three stages. MPPT buck charging, constant voltage absorption charging, and floating charge stage are the three charging phases.

Due of their simplicity, perturbation and observation approaches are employed. The approach has the benefit of just requiring two sensors and a straightforward circuit. By raising the voltage of a tiny PV array and tracking the power change, the algorithm was developed. The peak power point is caused by the disturbance if P is positive, and the operating point has shifted away from the peak point if P is negative. Perturbation must thus resume at its peak^6^.

de Oliveria^7^ claims that mismatching of phenomena, such as PSC, that PV arrays frequently experience, may be resolved using the particle swarm optimization (PSO) based MPPT approach. Since use of the MPPT techniques is taken to determine the dc-bus reference in order to ensure proper grid-tied inverter Operation, the effectiveness of PSO based MPPT technique is highlighted and compared with the P&O MPPT technique.

In contrast to traditional algorithms like the P&O algorithm or the IC method, the fuzzy logic controller (FLC) is one of the majority of algorithms that guarantees adequate performance MPPT in a variety of conditions, according to the author’s study^8^. After modelling the PV cell with the DC/DC converter and load, the suggested technique uses inputs to FLC to monitor the MPP, and its produced outputs are obtained as data files. Sixty-six percent of the stored data is used for training, while the remaining data is used for model testing. To represent the FLC, the structure of an artificial neural network (ANN) is specified. The backpropagation algorithm-based ANN training uses the data acquired from the FLC. The least mean square between the ANN and FLC models is calculated using this approach to identify the ideal parameters.

A voltage inverter is used to convert the direct current from the boost converter’s output to alternating current^9^. Here, the pulse width modulation (PWM) technique and the d-q frame control approach are utilised side by side to regulate the amount of current injected into the grid synchronous reference frame. Additionally, the inverter’s output needs to be coordinated with the grid. As a result, a Phase Locked Loop (PLL) approach is used to match the network’s frequency and phase. It is also advisable to employ an LC filter and transformer to create galvanic isolation and eliminate high frequency harmonics after the inverter.

Solar energy is converted by a photovoltaic array into DC voltage and current, which are controlled by a DC–DC boost converter that monitors P&O maximum power. The three-phase inverter uses an algorithm to track the location of the greatest field power before converting the DC voltage to AC for grid interface or local load power. A bandpass filter eliminates harmonics from the inverter’s output, and control circuits enable MPPT control, synchronization, and switching. A delta star transformer boosts output voltage and circulates zero sequences before connecting to the grid^10^.

MPPT controllers can be designed to work with any one of the methods for detecting the maximum power point. There are numerous techniques to track the maximum power point and selection of MPPT for any specific task depends upon several factors such as complexity of implementation. overall cost, response time, ability of the algorithm to detect the local and global maximum power point etc. Some methods to track the maximum power point are Perturb and observe (Hill climbing method), Incremental conductance method (IC), Fractional open-circuit Voltage (FOCV), Fractional short-circuit current (FSCC), Fuzzy Logic control, Neural Network, Sliding mode control (SMC), Robust unified control algorithm (RUCA), Particle swarm optimization technique (PSO), Grey-wolf optimization technique (GWO), Intelligent monkey king evolution (IMKE). The work done by various researchers is briefed in Table 1.

**Table 1:** Work done by various researchers in the concerned area.

Ref. no.	Objective	Technique and advantage	Drawbacks/purpose
^10^	This paper represents the design of a P&O algorithm based MPPT charge controller for a stand-alone 200W PV system	Perturb and observe (P&O) technique. This work involves the design of MPPT charge controller using DC/DC buck converter and microcontroller	A prototype MPPT charge controller is tested with a 200 W PV panel and lead acid battery. The results show that the designed MPPT controller improves the efficiency of the PV panel when compared to conventional charge controllers
^11^	Design of a 50 kW Solar PV Powered Charging Station for EV’s station using MPPT based controller is designed and simulated in MATLAB Simulink	The system is initially designed in PVsyst Software and the equipment selected from PVsyst is then selected in Simulink with their respective parameters and results in form of Voltage, power, current, and State of charge, etc. have been extracted in for of graph for all the major equipment	The system works satisfactorily under the given conditions and can be modified by adding protection and other components to obtain a more realistic result. The result found is satisfactory with a slight error in the filtering process. Since solar power generation and charging takes place every day
^12^	Topologies for interfacing supercapacitor and battery in hybrid electric vehicle applications	A hybrid energy storage system (HESS) comprising of battery and supercapacitor (SC) has been employed to resolve the issues faced by single storage systems used for electric vehicles (EVs) application. The battery ESS is able to provide better mileage, but unable to deliver a higher speed	Further works are needed to achieve improved topology alongside a robust control strategy to achieve higher power- sharing, better battery degradation mitigation, and flexibility while reducing the converter size, weight and providing bidirectional flow
^13^	Performance evaluation of series hybrid and pure electric vehicles using lead-acid batteries and supercapacitors	Different scenarios for both pure electric vehicles and series hybrid vehicles to explore the effect on battery parameters and fuel consumption by using supercapacitors with lead-acid batteries, by changing the parameters like capacitance value and battery charge capacity for optimal operation points, and by changing the Jeepney driving cycle into a regular route cycle	The simulations also indicate that the use of lead-acid batteries in conjunction with supercapacitors compared to just using lead-acid batteries help in the reduction of fuel consumption in series hybrid vehicles and improved battery voltage, charge, and current parameters for both pure electric vehicles and series hybrid electric vehicles
^14^	Using supercapacitors, which have inherently low ESR, allows a realizable and efficient storage system for these high peak currents, and thus the performance of the energy storage system is enhanced, the efficiency and operating time improved, and the battery life extended	Battery management for hybrid electric vehicles using supercapacitors as a supplementary energy storage system	It is expected that new emerging technologies will be implemented in the HEVs, UPS and other battery operated systems to enhance their performance. This would probably include supercapacitors which hold promise for reducing the high-voltage battery energy requirements
^15^	A real-time energy optimization management for electric vehicles with hybrid energy storage system (HESS) is presented in this paper. Energy management strategy coordinating lithium-ion battery and ultra-capacitor for electric vehicle	In this paper, the quadratic optimal control algorithm is used in electric vehicles with HESS. This method can avoid computational disaster. Furthermore, through the analysis of simulation results of MATLAB/Simulink and ADVISOR, the optimization method can effectively reduce the battery workload and energy consumption	The results of the two algorithms were analyzed, which verified the outstanding advantages of the quadratic optimization in improving the vehicle dynamic performance of the electric vehicle, increasing mileage and reducing the work burden
^16^	Based on the battery and supercapacitor voltage, seven operation modes of battery and capacitor cooperation are designed. The control strategy is redesigned to match the modes, in which the key control parameters are calibrated based on three standard driving cycles	Battery degradation minimization-oriented hybrid energy storage system for electric vehicles. A battery/supercapacitor hybrid energy storage system is developed to mitigate the battery degradation for electric vehicles	Using an independent driving cycle as the test cycle, the simulation result shows the battery degradation mitigation reached as high as 30%, larger than the majority of the prior HESS. This result indicates that the HESS topology and control strategy can be optimized together to realize a better battery degradation mitigation target

All the techniques have their own advantages and disadvantages. When we consider the practical scenario for a photovoltaic (PV) generation system (PGS) the occurrence of partially shaded condition is quite common and selection of MPPT technique is very important. Conventional MPPT techniques fails in tracking maximum power point, under partially shaded condition due to presence of the multiple peaks. Conventional MPPT techniques works well in uniformly shaded condition, which has a single maximum power point in the P–V curve. The inability of conventional MPPT algorithm to track the MPP during partially shaded condition is due to the fact that these algorithms are using “hill-climbing” principle for moving to the next operating point in the direction of power increase. If the P–V curve is having multiple peaks conventional MPPT algorithm may track only local MPP. In order to overcome this need to use artificial intelligence techniques such as PSO, IMKE, GWO, etc.

## Photo-voltaic cell modelling

The equivalent circuit of a solar cell may be represented by an electrical circuit. When describing a PV cell, two variables are frequently used. These are the short-circuit current (I_sc_) and the open-circuit voltage (V_oc_). In data sheet for the module, the PV module manufacturers provide these characteristics.

### PV cell equivalent circuit representation

**Figure 1:**
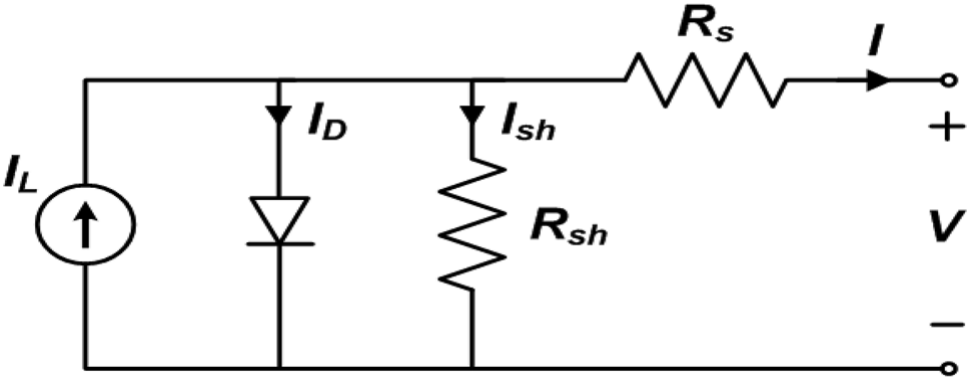
PV cell equivalent circuit.

Figure 1 represents the equivalent circuit of the PV cell. It consists of a current source which depicts the light generated current, a diode and a resistance connected in shunt across it. The array parallel resistance is denoted by R_sh_, and R_s_ which is the array series resistance. I_pv_ is the current produced by the incident light and it is directly proportional to the sun’s irradiance. I and V are the array’s output current and voltage.

The basic equations governing the I–V characteristics of PV cell are:

1 $$\mathrm{Iph }= \left[\mathrm{Isc }+\mathrm{ Ki }\left(\mathrm{T }- 298\right)\right]\times \frac{{\text{Ir}}}{1000},$$

2 $${\text{Irs}}=\frac{{\text{Isc}}}{\left[{\text{exp}}\left(\frac{{\text{qVoc}}}{{\text{NsknT}}}\right)-1\right]},$$

3 $$Io=Irs \times {\left[\frac{T}{Tr}\right]}^{3}{\text{exp}}\left[\left(\frac{{\mathrm{q \times Ego}}}{{\text{nk}}}\right)\left(\frac{1}{{\text{T}}} -\frac{1}{{\text{Tr}}}\right)\right],$$

4 $$I=Np \times Iph-Np \times IoX\left[{\text{exp}}\left(\frac{\frac{V}{Ns}+I \times \left(\frac{Rs}{Np}\right)}{n \times Vt}\right)-1\right]-Ish,$$

5 $$Vt=\frac{k \times T}{q},$$

6 $$Ish=\frac{V \times \left(\frac{Np}{Ns}\right)+I \times Rs}{Rsh}.$$

### Characteristics of PV array under un-shaded condition

**Figure 2:**
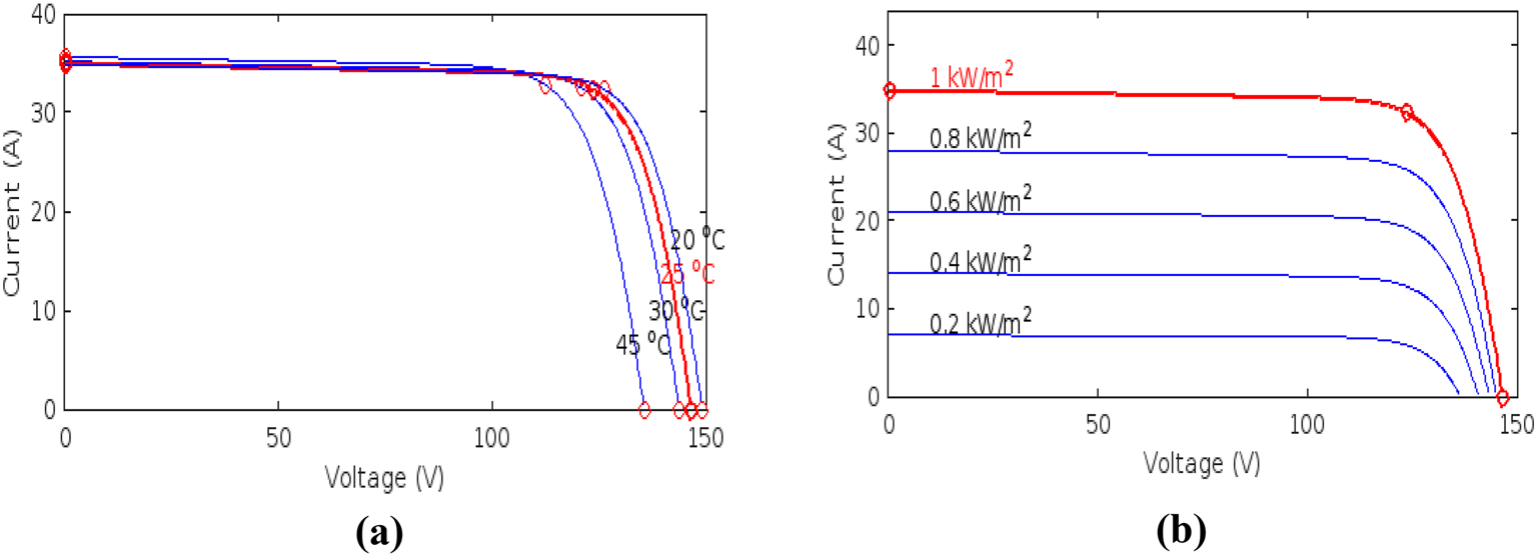
(**a**) Effect of temperature, (**b**) solar irradiance on I–V curves.

**Figure 3:**
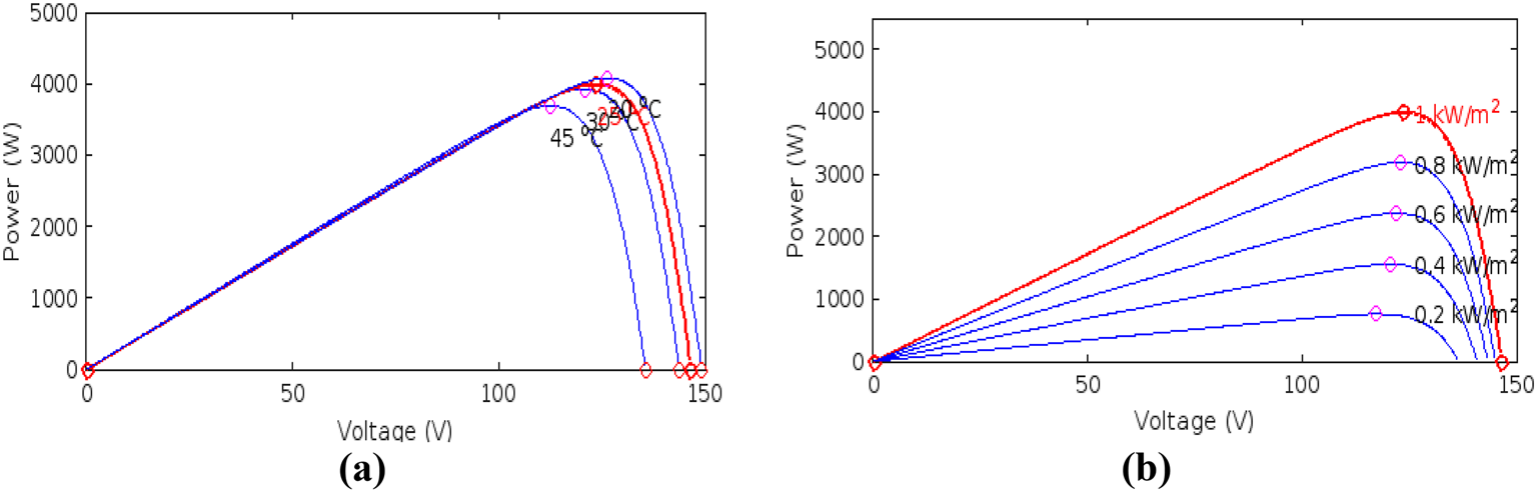
(**a**) Effect of temperature, (**b**) solar irradiance on P–V curves.

In un-shaded condition, the sunlight or solar insolation is uniform for all the PV modules in a PV array, so all the modules produce equal voltages. Photovoltaic cells have non-linear characteristics. The performance of PV cells as well as the output power is directly dependent on the change in the operating conditions (temperature & solar insolation). Figures 2 and 3 show the effect of change in the temperature and solar insolation on photovoltaic’s output current, voltage, and power.

## MPPT and battery charge controller technique

**Figure 4:**
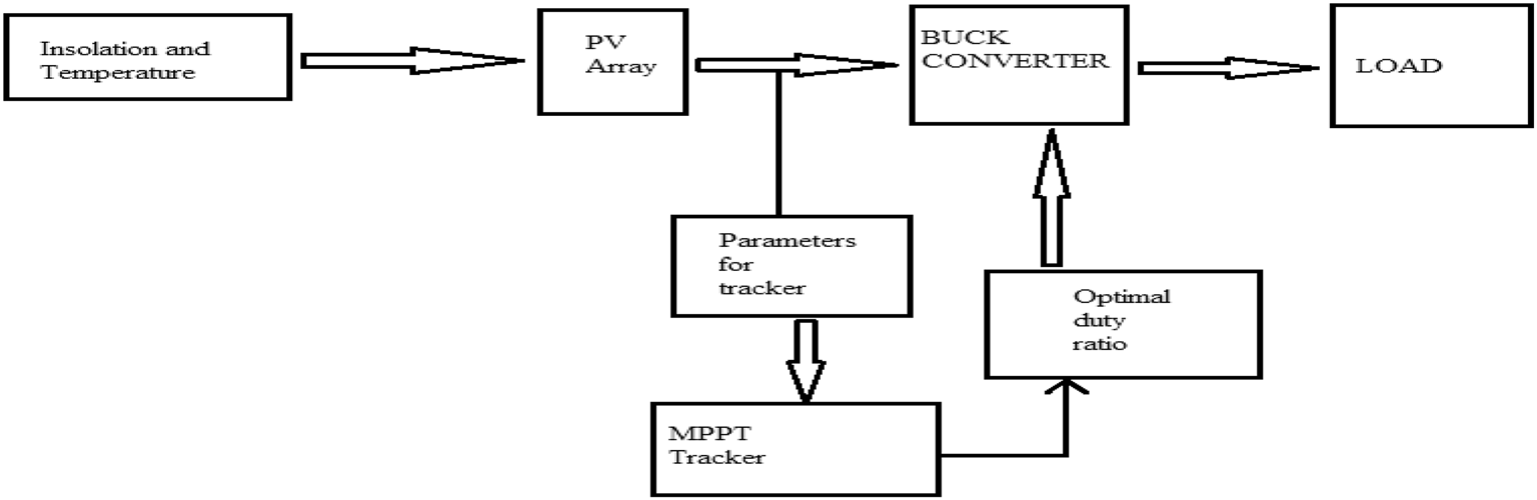
Block diagram of MPPT.

The PV characteristics of the PGS is nonlinear and highly influenced by solar insolation and temperature changes as shown in Figs. 2 and 3. This results in finding a reliable and efficient technique to adjust the photo-voltaic generating system operating point so that production of energy is maximized; it is indeed a challenging task. There exists only one terminal voltage for the PV array to operate with, for obtaining maximum power i.e. achieving the increased array efficiency. DC–DC SMPS converters plays very important role in MPPT tracking processes. As illustrated in Fig. 4, the DC-DC converters’ input terminals are linked to the output of the PV array, and the voltage of the array is controlled by varying the converter’s duty cycle while keeping the voltage at the maximum power.

### P&O (Perturb & Observe)

The Perturb & Observe (P&O) method is regarded as one of the most straightforward approaches of tracking Maximum Power Points. This is as a result of the processing time being relatively short. The phrase “hill-climbing” is another name for it. As the P–V curve’s peaks and troughs with regard to the maximum power point determine how it operates.

**Figure 5:**
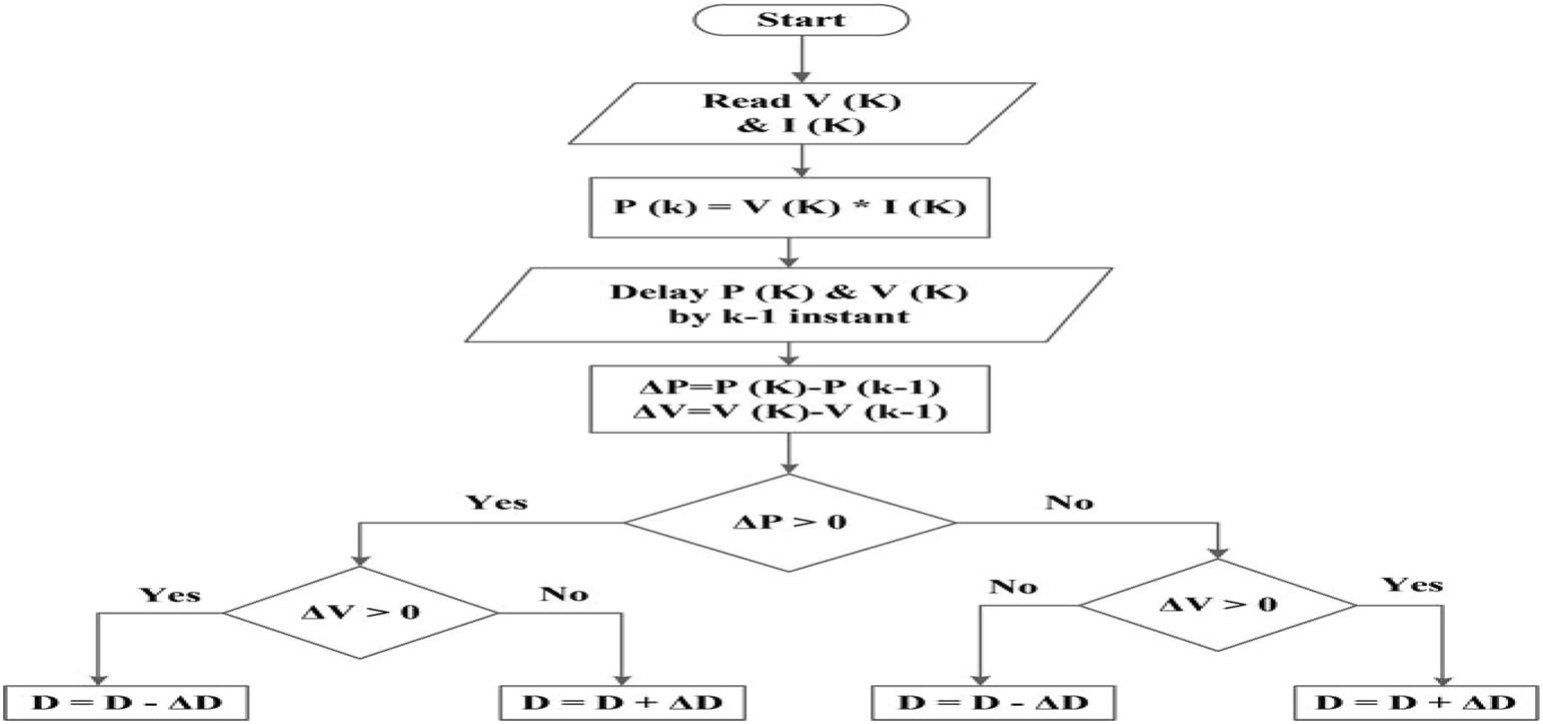
Flow chart of P&O algorithm.

P&O employs the sensors to detect the current and voltage of the solar PV array, as seen in Fig. 5. We can determine whether to raise or decrease the duty cycle using the MPPT method. deciding whether to increase or decrease the converter’s duty cycle in order to maximize power, we must compare the current measured power to the prior measured power. In order to maximize power, the boost converter’s duty cycle (D) is reduced if the input voltage is higher than the previous value. Reduce the duty cycle (D) if the input voltage is higher than the previous value to get closer to the MPP. The (P&O) then raises the duty cycle to monitor the maximum power point if the input voltage was lower than previously recorded and the input power was higher. Additionally, if D, or the perturbation, is sufficiently great, the oscillation will be endless and it will never reach its maximum power point. Without altering the physical characteristics of the solar panel, the P&O approach makes solar PV module calculations and design simpler.

### Battery charge controller

The lithium-ion battery charge controller was created to charge the battery in three stages. Three-stage charging consists of a trickle charging phase, constant voltage charging, and constant current charging. The first stage of constant current charging is also known as the bulk charging phase, and in this case, the charging current is at MPPT. The battery gets charged to its rated capacity at this stage. In the second step of constant voltage charging, sometimes referred to as the absorption charging phase, the battery is charged with a constant voltage; MPPT is not allowed at this stage. The final stage of floating charging simply keeps the state of charge (SoC) at 100% once the battery is fully charged. This prevents the battery from gassing and overheating from an unregulated overcharge to more than 100% Current.

**Figure 6:**
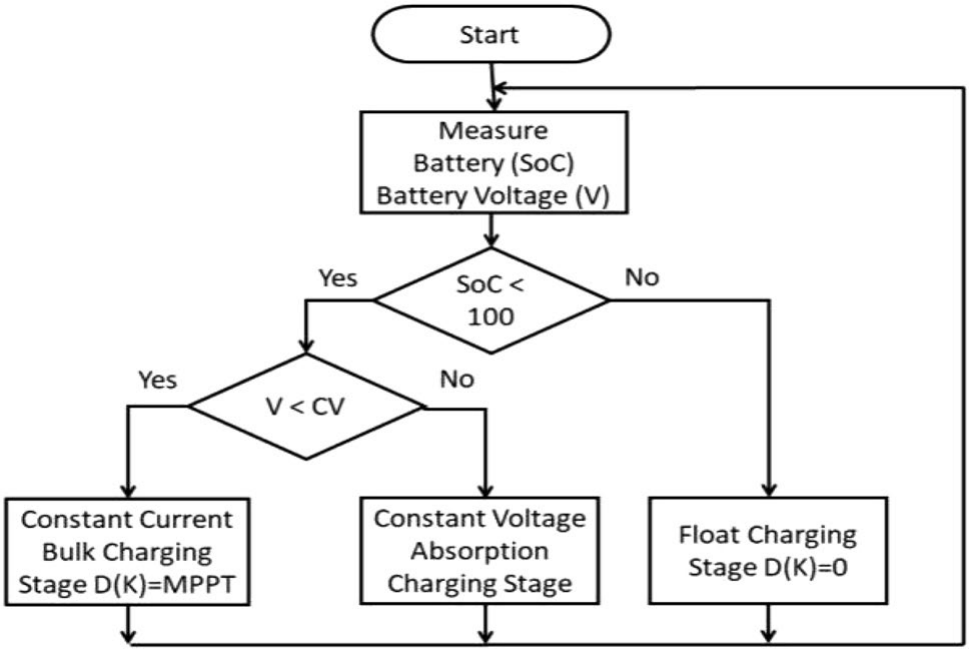
Battery charge controller flow chart.

A schematic of the battery charge controller is shown in Fig. 6. The charge controller measures the battery’s SoC and tension. If the SoC battery is below 100% in the first case, the charger enters the constant voltage or constant current charging phase; if not, it enters the floating stage, where the duty cycle D(K) is zero. According to the battery voltage level, the second scenario chooses either the bulk MPPT charging or constant voltage charging phase. If the battery voltage is below a constant set voltage value, the charger switches to the MPPT constant current phase of bulk charging; if not, it disables the MPPT and switches to the constant voltage absorption charging stage.

## System modeling

We utilized PVsyst for in-depth research, measurement, and data analysis of the photovoltaic system before utilizing MATLAB to follow the suggested approach to create a 4 kW PV-Powered charging station for EVs. Temperature: 25.3°C on average every year. Latitude/Longitude: 28.37° N/77.32° E.

The PVsyst report displays the latitude, longitude, altitude (205 m above sea level), and azimuth of the x-ray axis based on the data input into the PVsyst program, and the y axis shows the height of the sun. These locations allow us to determine the course of the sun. The image below shows that it will produce more energy during the summer (June) than during the winter (December). The average yearly solar radiation and midday temperature are shown in Table 2. The chart below shows how global radiation, solar radiation distribution, wind speed, and temperature change with the seasons. By examining the chart below, we can observe that each column in each month has the greatest pricing in (May, June, July, and August). An average of 250.7 kW/m^2^ is the annual worldwide radiation, which is 3264.4 kW/m^2^. Month denotes a 25.3 °C temperature.

**Table 2:** Monthly meter data^16^.

	Jan	Feb	March	April	May	June	July	Aug	Sept	Oct	Nov	Dec	Year
Horizontal globe (/kWh/m^2^)	88.9	112.0	158.3	174.7	183.6	170.8	148.0	144.2	141.0	124.1	93.8	87.5	1626.9
Horizontal diffuse (/kWh/m^2^)	50.9	56.3	71.5	83.9	101.6	101.9	96.7	92.6	84.5	73.3	57.8	51.9	922.9
Exteraterrestia (/kWh/m^2^)	192.6	210.7	278.0	309.4	344.0	341.1	348.1	329.5	284.9	248.7	197.0	180.3	3264.4
Cleanness index (ratio)	0.462	0.532	0.569	0.565	0.534	0.501	0.425	0.438	0.495	0.499	0.476	0.485	0.498
Ambient temp (°C)	13.3	17.6	23.7	29.7	33.5	33.1	31.4	30.4	29.2	26.5	20.2	15.0	25.3
Wind velocity (m/s)	1.8	2.1	2.2	2.4	2.6	2.6	2.4	2.1	1.9	1.3	1.2	1.4	2.0

### Matlab implementation of the block

**Figure 7:**
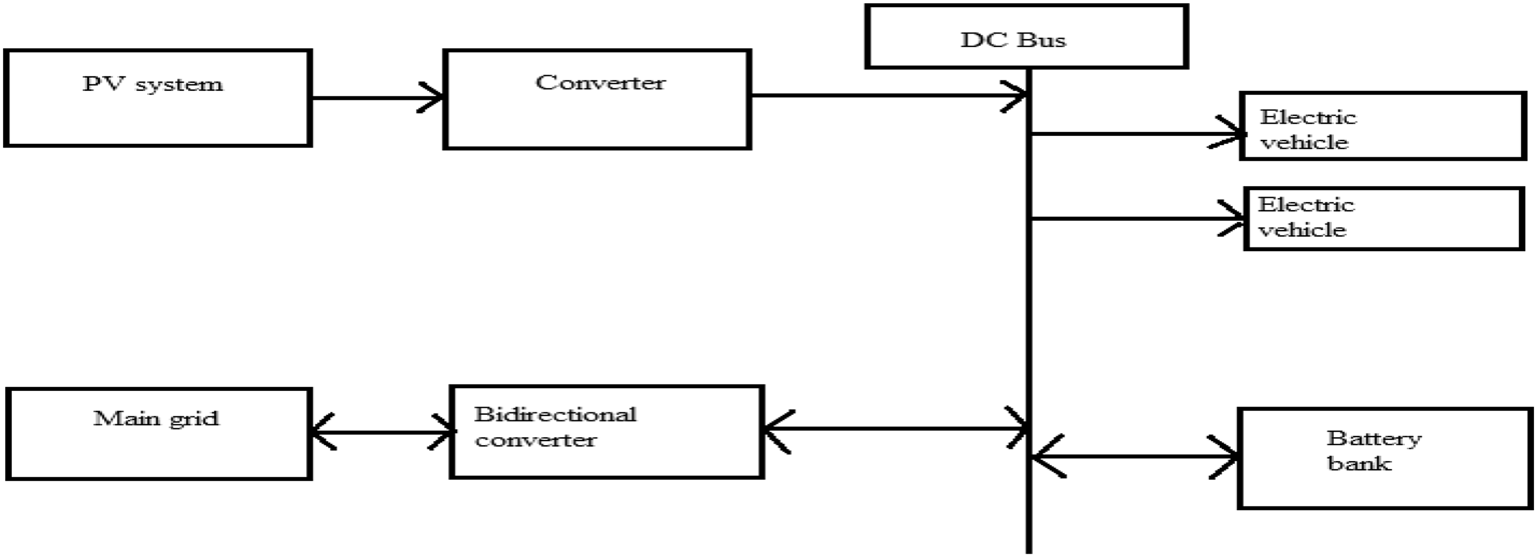
Matlab implementation block of hybrid charging station.

**Figure 8:**
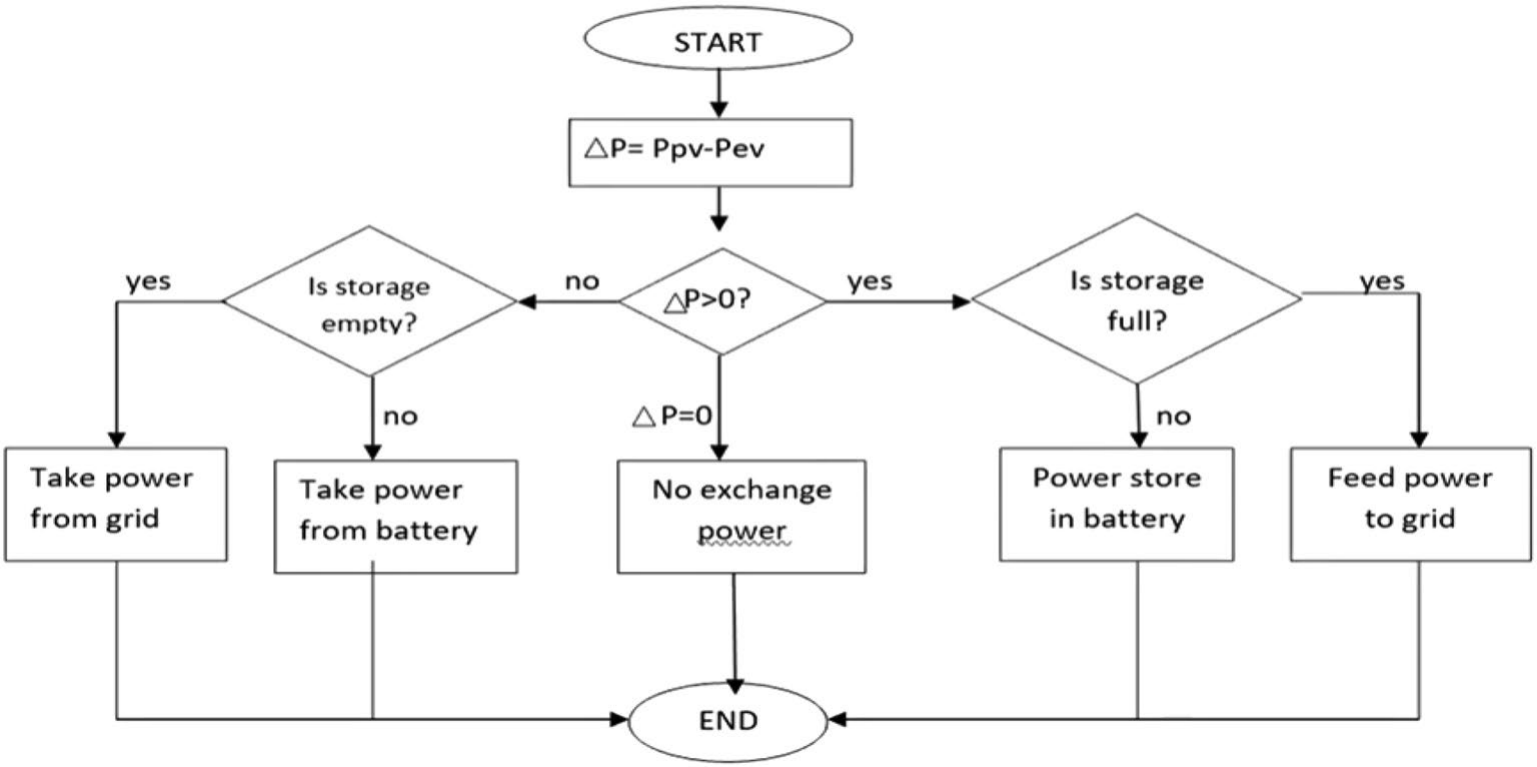
Flow chart of hybrid charging station.

Photovoltaic modules, electric vehicle charging stations (EVCS), battery banks, controllers, converters, connectors, cables, and mounting structures are the primary parts utilized in the described design. Block diagram for Matlab simulation and the flow chart of hybrid charging station is shown in Figs. 7 and 8 respectively.

### Mathematical calculations for power generation

Total power consumption demands by load:

$$\begin{aligned} {\text{Vehicle that want to charge }}\left( {\text{Yulu Bikes}} \right) & = {\text{ 48V}},{ 3}0{\text{ Ah}} \\ & = { 144}0{\text{ Wh}}/{\text{day}} \\ & = \, \left( {{\text{Total appliance watt}} - {\text{hours per day}}} \right) \times {1}.{3}{\text{.}} \\ \end{aligned}$$

Here, 1.3 is the factor loss in the system.

$$= { 144}0 \times {1}.{3 } = {\text{ 1872 Wh}}/{\text{day}}.$$

Suppose we are charging per day 10 vehicles then

$$= { 1872} \times {1}0 \, = { 1872}0{\text{ Wh}}/{\text{day}}.$$

Size of PV panel:

Suppose we will take here 5 h per day of panel generation factor.

$$\begin{aligned} {\text{So total power rating of panel }} = & {\text{ power consumption}}/{\text{panel generation}} \\ = & { 18,72}0/{5 } = {\text{ 3744 Watt}}{.} \\ \end{aligned}$$

Battery sizing of lithium-ion battery:

$${\text{Here vehicle want tot be charge }} = { 144}0 \times {1}0 \, = { 14},{4}00{\text{ Wh}}.$$

$${\text{Day of autonomy }} = {\text{ 1 day}},{\text{ Battery loss }} = \, 0.{85},{\text{ DoD }} = \, 0.{8}0,{\text{ Nominal voltage }} = {\text{ 48 V}},$$

$$\begin{aligned} {\text{Total battery Amp}} - {\text{hours }} = & \, [{144}00/0.{85} \times 0.{8} \times {48}] \, = { 441}.{176} \\ = & { 45}0{\text{ Ah approx}}.{\text{ 48 V}}. \\ \end{aligned}$$

### PVsyst report result with yearly generation and losses

**Figure 9:**
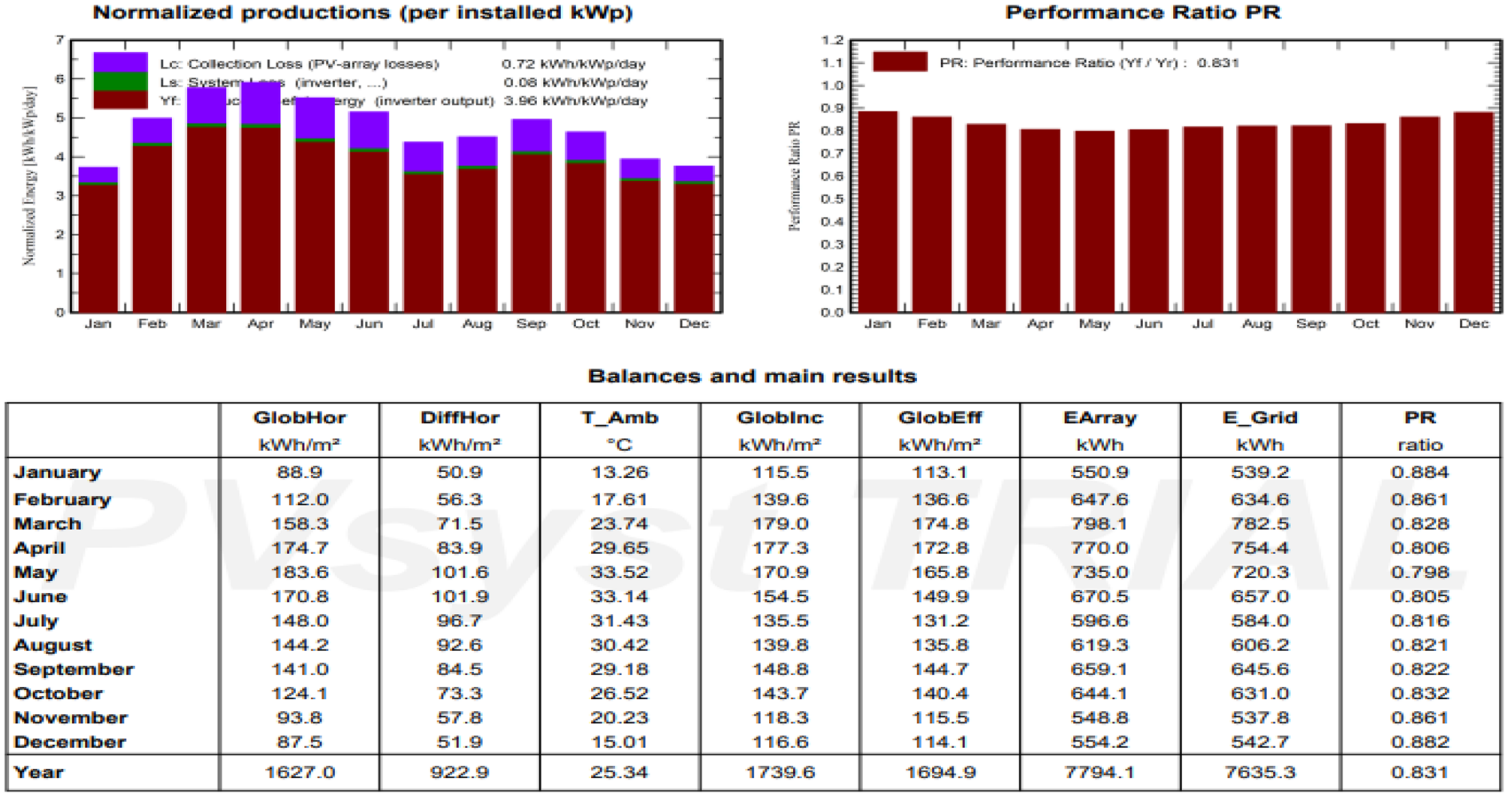
PVsyst report result. (PVsyst 7.3 is a PC software package for the study, sizing and data analysis of complete PV systems).

PVsyst report has been analyzed (Fig. 9) for given parameters and geo location on the basis of given inputs PVsyst report represents the Annual generation report with losses and generation factor of 3.96 and yearly generation of 7.6 MW.

Annual generation of 4 kW solar plant at give location is 7635.3 kW/year.

## Simulation results

**Figure 10:**
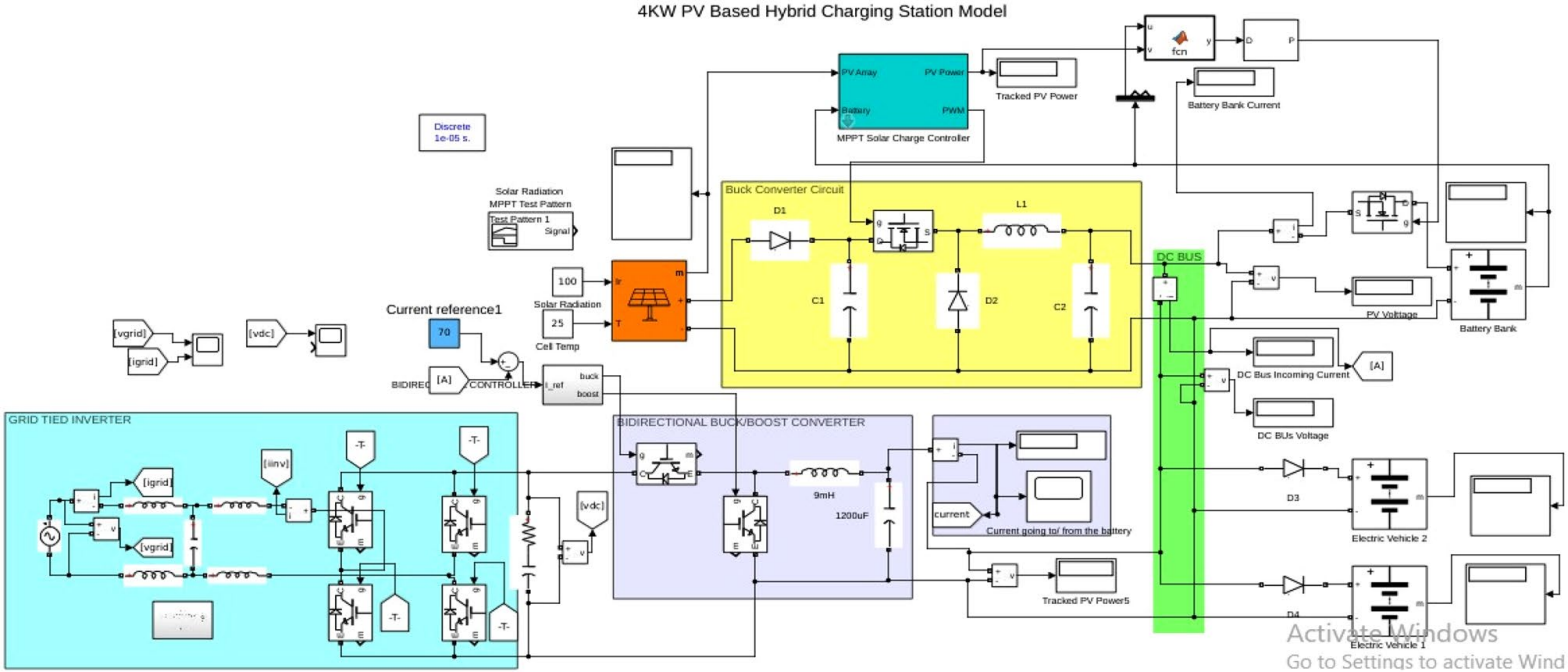
Hybrid charging station simulink model.

Eldora solar panels of 250W is used for simulation purpose. Electrical data sheet of SPR-E20-250 is shown in Table 3. 4 kW PV system configuration has 4 strings in parallel with 4 panels in each string. Simulation model consist separate blocks such as MPPT, battery bank, bidirectional buck/boost converter, grid tied inverter and EVs stand as shown in Fig. 10.

**Table 3:** Electrical data sheet of SPR-E20-250 (PV panel).

Battery bank type and rating	Lithium-ion, 48 V, 450 Ah
EV battery rating	48 V, 30 Ah
Nominal power (P_nom_) of PV panel	250 W
Total nos. of PV panels	16
PV panel configuration	4 × 4 (4 panels in one string and 4 strings)
Power tolerance	+ 5–0%
Average panel efficiency	20.4%
Rated voltage (V_mpp_)	30.2 V
Rated current (I_mpp_)	8.27 V
Open-circuit voltage (V_oc_)	36.6 V
Short-circuit current (I_sc_)	8.75 A
Maximum system voltage	1000 V IEC and 60 V UL
Maximum series fuse	15A
Power temperature coefficient	− 0.35%/°C
Voltage temperature coefficient	− 176.6 mV/°C
Current temperature coefficient	2.6 mA/°C

Simulation block runs in five different modes, these modes are as follows:

- Mode 1 (battery bank charging by PV System).
- Mode 2 (EVs charging by PV system).
- Mode 3 (EVs charging by grid when PV power is not enough).
- Mode 4 (EVs charging from battery bank when grid and PV system both are not available).
- Mode 5 (PV system feed power to grid).

### 4 kW PV system MPPT/charge controller waveforms

**Figure 11:**
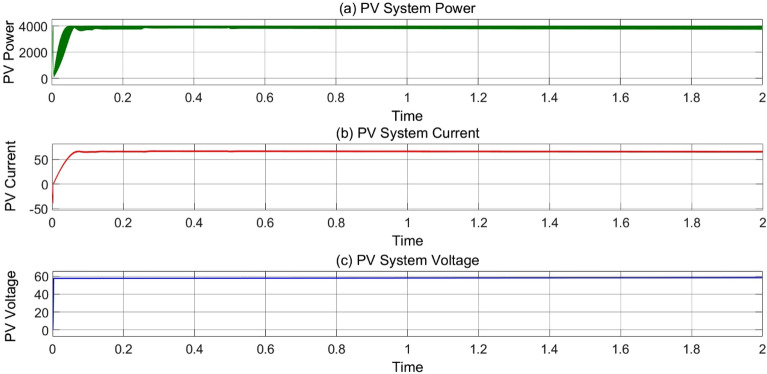
(**a**) Variation of MPPT track power (**b**) MPPT current and (**c**) MPPT voltage w.r.t time at 25 °C, 1000 Wb IR.

In Fig. 11a, the power production by PV grid is shown at 1000 W/m^2^ and 25 °C. The initial ripple is due to start of PV-panels and PI-controller. In Fig. 11b, The PV current is shown that reaches at constant value of 70 A for maximum power output after 0.5 s. Figure 11c shows the constant voltage of 54 V across DC bus to charge EV’s battery of 48 V.

### MODE 1 (battery bank charging by PV system)

**Figure 12:**
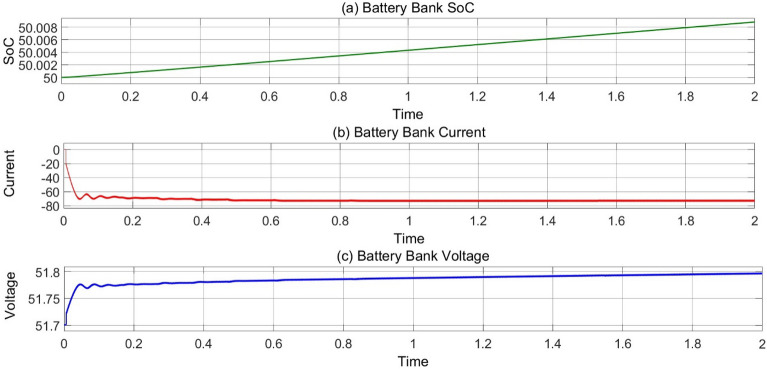
(**a**) Variation of battery SoC, (**b**) current and (**c**) voltage w.r.t time at 25 °C, 1000 Wb IR.

In Fig. 12, The EV’s charging SoC, current and voltage are representing in mode 1 operation when PV system charging the EV’s as load currently constant voltage of 54 V across DC bus is applied to charging the EV’s and graph represents the increment in battery’s SoC and Voltage and Charging current is constant.

### MODE 2 (EVS charging by PV system)

**Figure 13:**
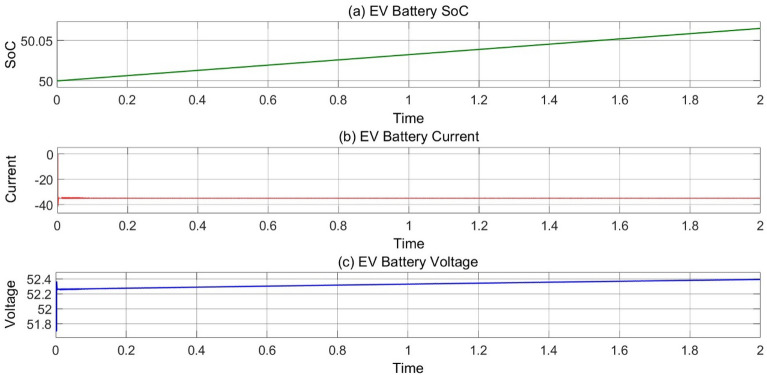
(**a**) Variation of EV’s battery SoC, (**b**) current and (**c**) voltage w.r.t time at 25 °C, 1000 Wb IR.

In Fig. 13, the EV’s charging SoC, current and voltage are representing in mode 2 operation.

### MODE 3 (EVS charging by grid when PV power is not enough)

**Figure 14:**
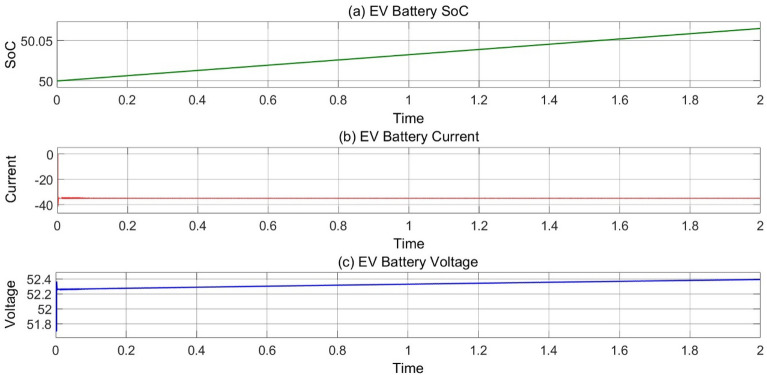
(**a**) Variation of EV’s battery SoC, (**b**) current and (**c**) voltage w.r.t time at 25 °C, 1000 Wb IR.

Figure 14 represents the mode 3 operation when EV’s are charging during night or rainy season when PV power is not enough to charge the EV’s. During this situation EV’s takes charging through AC grid which have a bidirectional inverter and bidirectional buck-boost converter.

### MODE 4 (EVS charging from battery bank when grid and PV system both are not available)

**Figure 15:**
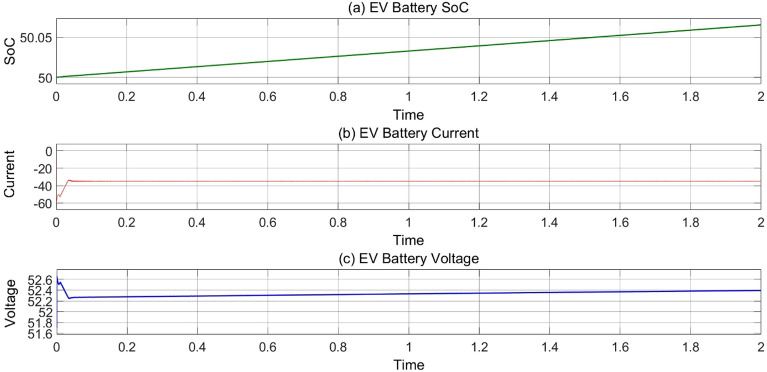
(**a**)Variation of EV’s battery SoC, (**b**) current and (**c**) voltage in mode 4 operation.

In Fig. 15, Mode 4 operation graphs represent the charging condition of EV’s through Battery bank. This operation works in long power cut fault. When PV system and AC grid both are not available at same time so EV’s are charging through battery bank which have power backup.

### MODE 5 (PV system feed power to grid)

**Figure 16:**
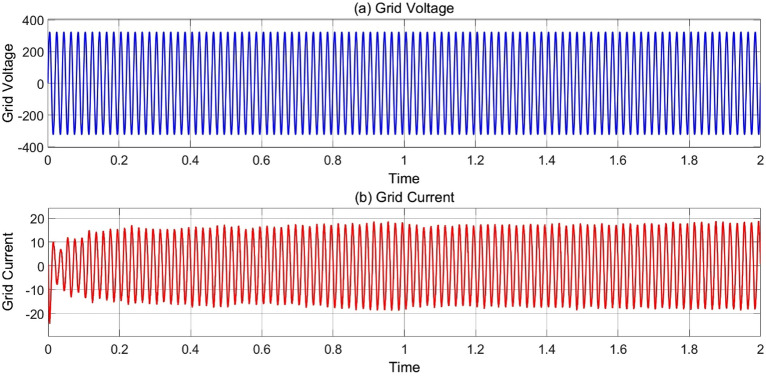
(**a**) Variation of voltage and (**b**) current graph w.r.t time at 25 °C, 1000 Wb IR.

When the charging station have no load as EV’s and Battery bank is also full charged this time PV system generate power and feed to the grid which also helps in earning and balancing the grid load during peak hours. The graph of Voltage and current feed to the AC grid are shown in Fig. 16.

The simulation results of the 5 different modes of operation for the EV charging station have been validated through the use of MATLAB and PVsyst. The modes include:

- Mode 1: battery bank charging by PV system.
- Mode 2: EVs charging by PV system.
- Mode 3: EVs charging by grid when PV power is not enough.
- Mode 4: EVs charging from battery bank when grid and PV System both are not available.
- Mode 5: PV system feed power to grid.

The simulation results demonstrate the effectiveness of the hybrid charging station in providing uninterrupted power for EVs. The three-stage charge controller, buck converter, grid-tied inverter circuit, and MPPT P&O tracking algorithm have been shown to be entirely replicable. The system is capable of charging 10–12 EVs with 48 V 30 Ah lithium-ion batteries, and it can export surplus solar energy to the grid, reducing the load demand. Additionally, the simulation results show the operation of the charging station in different scenarios, such as during the night or rainy season when PV power is not enough, and during long power cuts when both the grid and PV system are unavailable.

The simulation results validate the effectiveness of the hybrid charging station in addressing the challenges associated with grid stability and EV charging, and contribute to the advancement of sustainable transportation infrastructure and renewable energy integration. The system’s ability to integrate solar power and battery energy storage to provide uninterrupted power for EVs is a significant step towards reducing reliance on fossil fuels and minimizing grid overload.

## Conclusion

Simulink modelling of a charging controller and a detailed hybrid charging station is provided. The three-stage charge controller, buck converter, grid-tied inverter circuit, and MPPT P&O tracking algorithm are all discussed in detail and are entirely replicable. By keeping track of the maximum output from the 4 kW PV field energy source and regulating the charge using a three-stage charging strategy, the 4 kW PV-based charging station is capable of charging 10–12 EVs with 48 V 30 Ah lithium-ion batteries.

The system was first created in PVsyst. Following the selection of software and devices using parameters from PVsyst in Simulink, results in the form of voltage, power, current, and state of charge, among other metrics, have been extracted into graphs for all significant devices. The filtering process could be optimized, but the overall result is satisfactory. The research contributes to the advancement of sustainable transportation infrastructure and renewable energy integration, addressing the challenges associated with grid stability and EV charging.

## Data Availability

Data may be available upon reasonable request from the corresponding author.
